# How Oral Medicine Practice Is Reported: A Scoping Review of 114,971 Patients

**DOI:** 10.1111/odi.70017

**Published:** 2025-07-02

**Authors:** Camila Barcellos Calderipe, Laura Borges Kirschnick, Alini Cardoso Soares, Ana Carolina Uchoa Vasconcelos, Marcio Ajudarte Lopes, Nathaniel Simon Treister, Alan Roger Santos‐Silva

**Affiliations:** ^1^ Oral Diagnosis Department, Piracicaba Dental School University of Campinas (UNICAMP) Piracicaba São Paulo Brazil; ^2^ Department of Operative Dentistry Faculty of Pharmacy, Dentistry and Nursing, Federal University of Ceará (UFC) Fortaleza Ceará Brazil; ^3^ Division of Oral Medicine and Dentistry Brigham and Women's Hospital Boston Massachusetts USA; ^4^ Department of Oral Medicine, Infection and Immunity Harvard School of Dental Medicine Boston Massachusetts USA

**Keywords:** oral medicine, scope of practice, stomatology

## Abstract

**Objective:**

This review aimed to: (i) identify the variables used by oral medicine services (OMS) to describe the scope of their clinical practices and (ii) identify gaps in the availability of these variables in the literature to guide future research on the characterisation of OM practices. To address the following question: What characteristics from studies on clinical practice have been used to describe the scope of practice in OM?

**Material and Methods:**

This scoping review followed the PRISMA‐ScR guidelines. Electronic searches were conducted in the following databases: PubMed, Scopus, Embase, Web of Science, LILACS and grey literature. The presence of the following variables was assessed: referral source, age, sex, medical profile, diagnosis, procedures and follow‐up.

**Results:**

A total of 12 studies met the eligibility criteria, comprising 15 OMS across 10 countries and including 114,971 patients. Some studies specifically aimed to characterise OMS practice, while others focused on specific aspects of these practices. Most studies examined variables such as referral source, age, sex and diagnosis.

**Conclusions:**

There are gaps in the description of the OMS scope of practice in the literature, particularly regarding patient follow‐up, characterisation of the range of procedures performed and documentation of patients' medical profiles.

## 1 Introduction

Oral medicine (OM), referred to as Stomatology in Ibero‐American countries and Southern Europe, has existed as a distinct clinical field since the 1940s (Scully et al. 2016). The field is expanding, having been well established for years in some regions, while it is just now being recognised in others (Rogers et al. 2011; Bez et al. 2017; Stoopler and Murdoch‐Kinch 2020; Santos‐Silva et al. 2022). The scope of OM practice is broad, encompassing the management of patients with complex systemic conditions and a wide range of clinical diagnoses, which require multiple therapeutic interventions (Miller et al. 2001). The most common settings for OM practice are dental schools and hospitals, although it also occurs in private clinics, public health clinics and medical schools (Stoopler et al. 2011).

Studies have shown that patients typically consult more than two healthcare professionals on average before being referred to an OM clinician (Miller et al. 1997; Pinto et al. 2015). Considering that OM frequently encompasses chronic, severe and painful conditions that can significantly impact the quality of life (Scully et al. 2016), it is essential for OM to be widely recognised to ensure timely referrals. Thus, this review aimed to: (i) identify the variables used by oral medicine services (OMS) to describe the scope of their clinical practices and (ii) identify gaps in the availability of these variables in the literature to guide future research on the characterisation of OM practices. To address the following question: What characteristics from studies on clinical practice have been used to describe the scope of practice in OM?

## 2 Material and Methods

### 2.1 Protocol and Registration

This scoping review was conducted in accordance with the guidelines of the Preferred Reporting Items for Systematic Reviews and Meta‐Analyses extension for Scoping Reviews (PRISMA‐ScR) (Tricco et al. 2018). A protocol was developed and registered on the Open Science Framework (OSF) at the following DOI: https://doi.org/10.17605/OSF.IO/UAPWZ.

### 2.2 Eligibility Criteria

The inclusion criteria were established using the PCC acronym (Population, Concept and Context) as follows: (P) OMS; (C) studies characterising the scope of practice in OM; and (C) university, hospital, private or public clinic. The sources of evidence were retrospective studies.

The exclusion criteria were as follows: (1) aggregated data from multiple OMS without the possibility of distinguishing between them; (2) exclusive retrospective analysis of histopathological findings; (3) studies focusing on a specific group of diseases and/or age groups; (4) studies aimed solely at describing the prevalence of OM diagnoses; and (5) studies for which full texts were unavailable.

### 2.3 Information Sources and Search Strategy

Personalised search strategies were conducted on 3 August 2024, for the following databases: PubMed/MEDLINE (National Library of Medicine), Scopus (Elsevier), Embase (Elsevier), Web of Science (Clarivate Analytics/Thomson Reuters) and LILACS (Virtual Health Library). Grey literature was searched through Google Scholar and ProQuest (File S1). Additionally, manual searches were performed using the references of the selected articles.

### 2.4 Selection Process

The articles identified were imported into the EndNote reference manager, where duplicates were removed, and subsequent screening was conducted. The titles and abstracts of all identified articles were read independently by two authors (C.C. and L.K.). If the title and abstract met the eligibility criteria, the reference was selected for full‐text review. After evaluating the full text, articles that met the inclusion criteria were selected. Divergent opinions were resolved through consensus between C.C. and L.K.

### 2.5 Data Extraction

The following data were assessed in each included article, when available: study characteristics (author; year of publication; country); service and service setting; sample size; period of sample collection (in years); objective; conclusions; presence and absence of the following variables: referral source, age (mean and range), sex, comorbidities, harmful habits, diagnosis, procedures (diagnostic and therapeutic) and follow‐up. Age was considered ‘unclear’ when the mean age of the sample was not reported. The diagnosis was considered ‘unclear’ in the following cases: when only the clinical feature of the lesion was described (e.g., white lesion); when the diagnosis was reported as a collective category; when a study included two samples from two different OMS and the diagnoses were not clearly attributed to each OMS; or when only the percentage of diagnoses was provided, without the corresponding absolute number. Diagnostic procedures were considered ‘unclear’ when: two samples from two OMS were included in a study and the indication of procedures was not clear for the different OMS; when the examination request was made outside of the OMS; or when only the percentage of diagnostic procedures was reported, without the corresponding absolute number. The therapeutic procedure was considered ‘unclear’ when only one medication was mentioned or when the absolute number of therapeutic procedures was not provided, with only the percentage presented. Follow‐up was considered ‘unclear’ when there was limited information on patient return visits records.

Additionally, when available, an overview of the prevalence highlights of the previously established variables was provided. Lastly, other variables that were not the focus of our investigation but were present in the studies were mentioned at the end of the Results section, in order to demonstrate additional target data from the studies.

### 2.6 Synthesis of Results

The data were synthesised descriptively.

Graphical representations were created using the Matplotlib library (developed by the Matplotlib Development Team, Chicago, IL, USA), a Python‐based data visualisation tool.

## 3 Results

### 3.1 Study Selection

**FIGURE 1:**
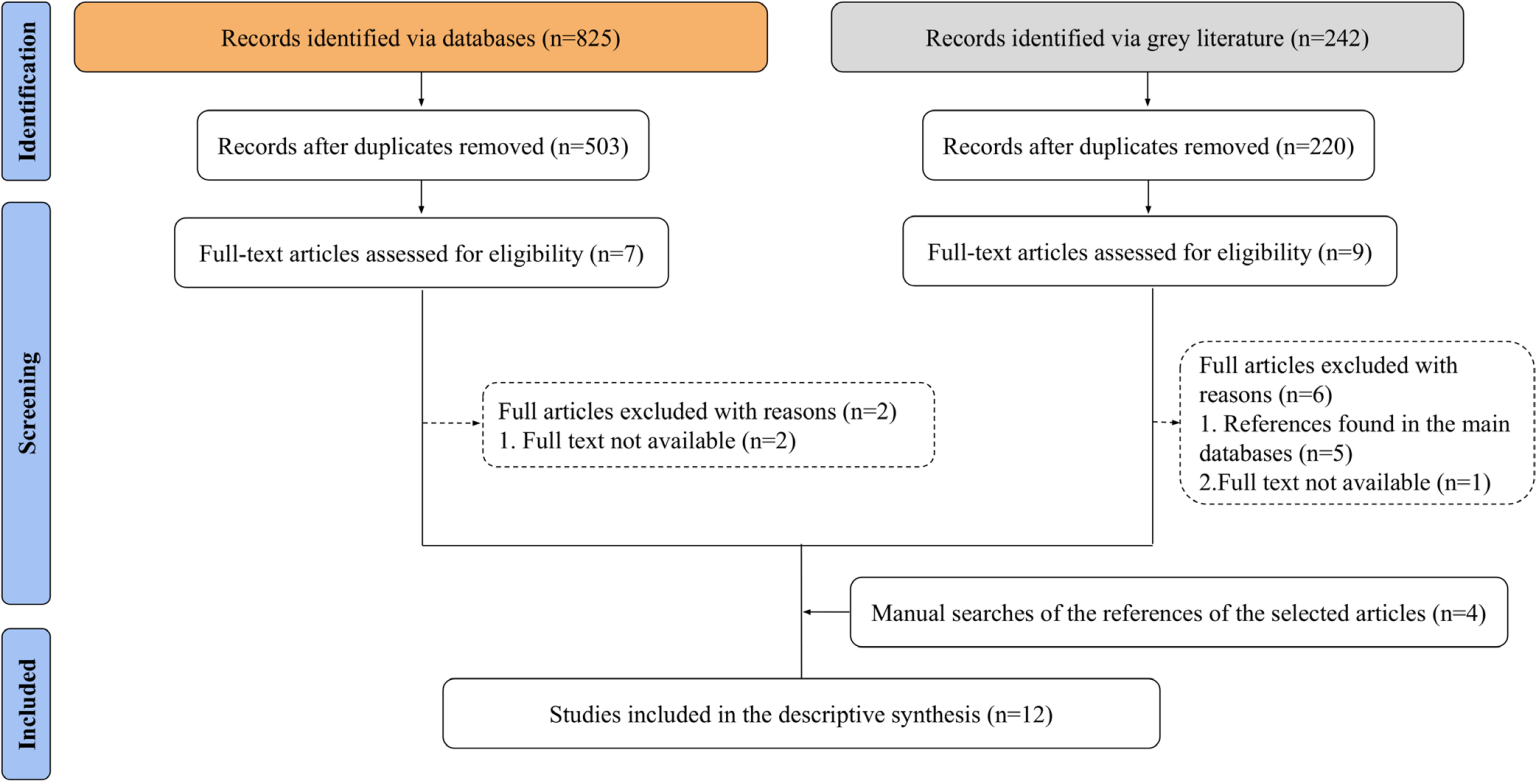
Flow diagram of literature search adapted from PRISMA (2020).

The searches identified 825 articles in the five electronic databases and 242 records in the grey literature, totaling 1067 records. After removing 344 duplicate references, 723 remained, with 503 from the main databases and 220 from the grey literature. The titles and abstracts were then screened, resulting in 16 articles selected for full‐text review. Of these, 8 were excluded for the following reasons: full text not available (*n* = 3) and duplicates found in the main databases (*n* = 5). Additionally, manual searches of the references from the selected articles identified four more articles for inclusion. In total, 12 studies met the eligibility criteria, representing 15 OMS and encompassing 114,971 patients (Figure 1).

### 3.2 General Characteristics of the Included Studies

The included articles were published between 1990 and 2022. Most of the OMS were from North America (*n* = 6/40.0%), followed by Oceania (*n* = 3/20.0%), Asia (*n* = 2/13.3%), Europe (*n* = 2/13.3%), Central America (*n* = 1/6.6%) and South America (*n* = 1/6.6%). OMS settings included dental schools (*n* = 7/46.6%), hospitals (*n* = 5/33.3%), private clinic (*n* = 1/6.6%), medical school (*n* = 1/6.6%) and school of medicine and dentistry (*n* = 1/6.6%). The mean sample size in dental school–based settings was 1004.5 patients (range: 106–2533), with a mean analysis period of 4 years (range: 1–8). The mean sample size in hospital‐based settings was 21,065.6 patients (range: 378–97,070), with a mean analysis period of 4 years (range: 1–6). In the other settings, the mean sample size was 870 patients (range: 583–1104), with a mean analysis period of 2.61 years (range: 1–5) (Table 1; File S2).

**TABLE 1:** General features of the included studies.

Variables	*n*	%
Continent (*n* = 15)		
North America	6	40.0
Oceania	3	20.0
Asia	2	13.3
Europe	2	13.3
Central America	1	6.6
South America	1	6.6
Settings (*n* = 15)		
Dental schools	7	46.6
Hospitals	5	33.3
Private clinic	1	6.6
Medical school	1	6.6
School of medicine and dentistry	1	6.6
Mean sample size (*n* = 15)		
Dental schools	1004.5 (106–2533)	
Hospitals	21,065.6 (378–97,070)	
Other settings	870 (583–1104)	
Mean analysis period in years (*n* = 15)		
Dental Schools	4 (1–8)	
Hospitals	4 (1–6)	
Other settings	2.61 (1–5)	

### 3.3 Assessment of the Availability of Variables From Oral Medicine Services

**FIGURE 2:**
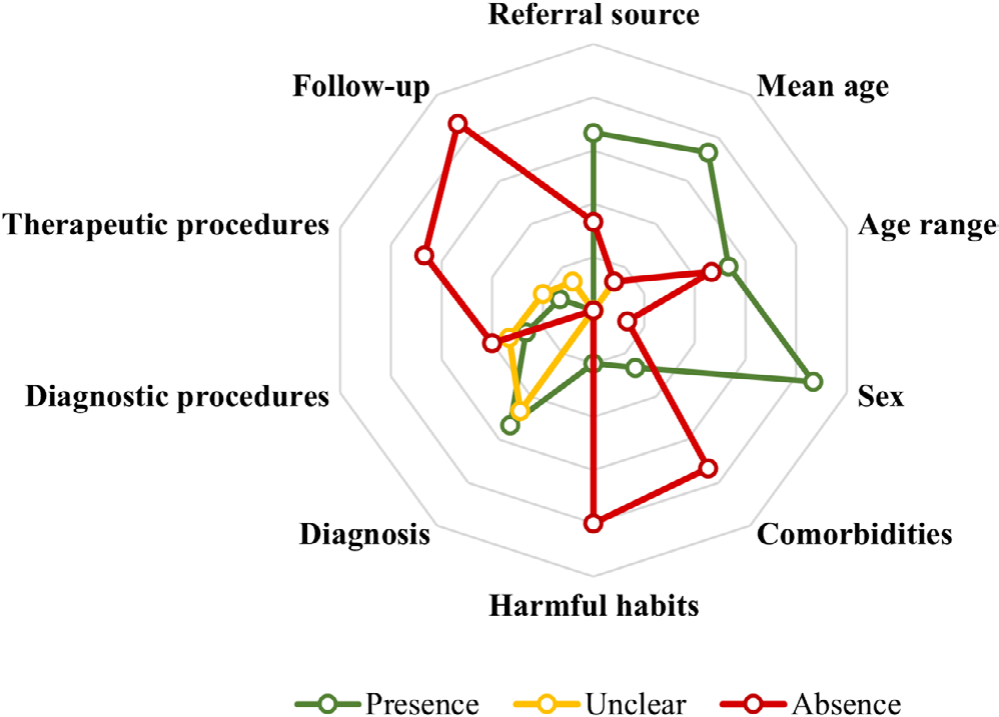
Availability of data from OMS. The radar chart illustrates the presence, absence and unclear form of the data analysed in the included studies. The rays represent percentages, with the outermost ray indicating 100%, and the inner gradations reflecting reductions of 20% down to the centre, which represents 0%.

**FIGURE 3:**
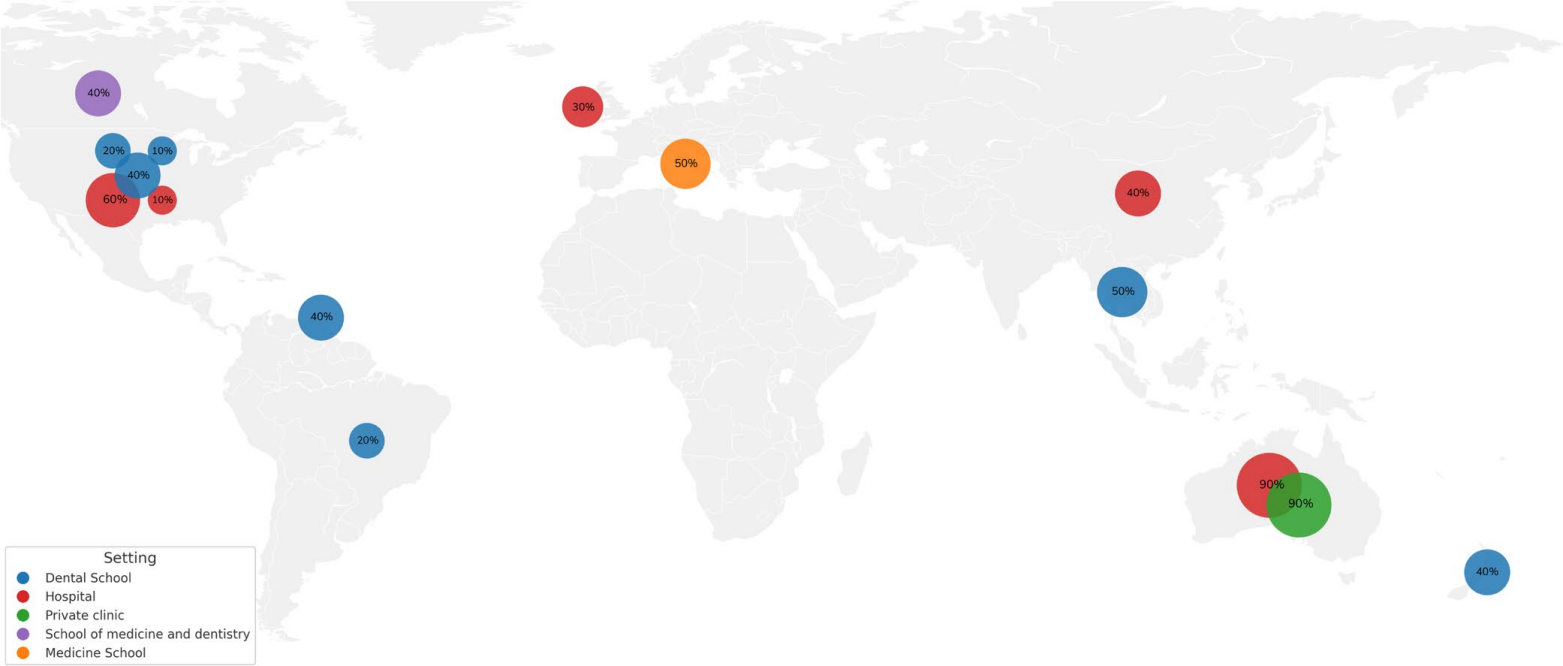
Bubble map illustrating the individual OMS included in the review. Each bubble represents a service and its size reflects the percentage of predefined variables available in that specific study. These variables include referral source, age reported as mean and range, sex, comorbidities, harmful habits, diagnosis, diagnostic and therapeutic procedures, and follow‐up. Therefore, the percentage shown within each bubble corresponds to the presence of these variables reported in each study.

The patient referral source was assessed in 10 (66.7%) OMS. The mean age was reported in 11 (73.3%) studies and unclear in 2 (13.3%) studies, while the age range was reported in 8 (53.3%) studies. Patient sex was reported in 13 (86.7%) studies. Comorbidities were assessed in 4 (26.7%) services, and harmful habits were reported in 3 (20.0%) samples. Diagnoses were reported in 8 (53.3%) OMS, and unclear diagnoses were found in 7 (46.7%) studies. Diagnostic procedures were described in 4 (26.7%) samples, and unclear procedures were reported in 5 (33.3%) OMS. Therapeutic procedures were reported in 2 (13.3%) OMS and were unclear in 3 (20.0%) OMS. Patient follow‐up was unclear in 2 (13.3%) services (Figure 2; File S3). The presence of the variables analysed within each service is presented in a bubble map in Figure 3. Each bubble represents a service, and its size reflects the presence of the variables reported in the corresponding study.

When available, an overview of the investigated variables was provided qualitatively, based on the highest prevalence reported in each article (File S4).

In addition to the previously established and evaluated variables in this review, other variables were collected in the included studies, including: number of doctors seen prior to OMS referral; referring physician's specialty; years of experience of the referring clinician; patient's place of residence (within or outside the city); time between referral and consultation; distance travelled by the patient (miles/km); TYPE of health insurance; ethnicity; reason for referral; medication history; and location of the lesion/condition.

## 4 Discussion

The findings of this original scoping review, based on data from 15 OMS located in 10 countries and covering a population of 114,971 OM patients, revealed that some studies specifically aimed to characterise the practice in OMS, while others focused on aspects of these practices. However, gaps were identified in the reporting of OMS practices, particularly regarding information on patient follow‐up, the range of diagnostic and therapeutic procedures, and documentation of patients' medical profiles.

Enhancing this body of evidence could contribute to a broader recognition of OM as a vital clinical specialty. Studies conducted nearly two decades apart showed that, on average, patients consulted more than two healthcare professionals before being attended by an OM professional (Miller et al. 1997; Pinto et al. 2015). These findings are consistent with more recent data, which revealed an average of 1.9 consultations before referrals to an OMS (Coppola et al. 2021). One possible consequence of the insufficient recognition of OM is the low density of professionals and the uneven geographical distribution (Yoon et al. 2024). These factors likely contribute cyclically to the lack of recognition of the field and delays in referrals.

The American Academy of Oral Medicine (AAOM) defines OM as ‘The specialty of dentistry responsible for the oral health care of medically complex patients and for the diagnosis and management of medically related disorders or conditions affecting the oral and maxillofacial region’ (American Academy of Oral Medicine Oral Medicine History 2024). However, there are variations in the definition and scope of practice of this specialty from country to country (Rogers et al. 2011; Bez et al. 2017; Stoopler and Murdoch‐Kinch 2020; Santos‐Silva et al. 2022), reflecting the heterogeneous development of OM worldwide (Esteves‐Pereira et al. 2025). Although this was not the primary aim of this study, creating a global catalogue of the scope of OM practice would be beneficial in educating both patients and healthcare professionals about the range of services provided by OMS. In this regard, more standardised studies characterising the scope of practice in local OMS would help foster a global understanding of OM practice in the future.

In the present review, most OMS samples were located in the United States, which aligns with the fact that the country was a pioneer and maintains a prominent role in the growth of the field (Scully et al. 2016). Although this finding is specific to the studies included, it may also reflect a broader trend of disproportionate geographic distribution of OM scientific organisations across continents (Esteves‐Pereira et al. 2025). OMS are more frequently found in dental schools, followed by hospital settings (Stoopler et al. 2011), as reflected in this review. OM is still in the process of being integrated into the healthcare system, and it is expected that the growing recognition of its services will contribute to increasing and expanding its presence (Yoon et al. 2024). The mean period in years for the samples from dental schools and hospital settings was the same. However, the mean number of patients in the sample exhibited a notable difference, which is naturally related to the considerably higher patient flow in hospitals compared to dental schools. Overcoming the recognition barrier in OM is crucial for improving care and health outcomes. In this regard, how OMS demonstrate their practices through retrospective studies could play a significant role. For example, collecting data on the referral source helps in identifying which professionals are collaborating with OMS and provides insight into the level of knowledge of patients who present without a prior referral. Furthermore, the multidisciplinary nature of OM contributes to a wide range of referral pathways. A prime example of this close relationship is the need for collaboration with oncologists to prevent/treat oral toxicities related to cancer treatment (Watters et al. 2011; Bhandari et al. 2020; Ruggiero et al. 2022). However, this remains a challenge; Aljishi et al. (2024) found that 54% of oncologists in the United States were not familiar with the OM specialty (Aljishi et al. 2024), a problem that persists within the medical profession in general, as shown in other studies (Alrashdan et al. 2019; Almazrooa and Binmadi 2021). Study designs demonstrating the integration of interdisciplinary management approaches (e.g., Oncology, Rheumatology, or Dermatology) could provide valuable insights into the broader context of OM practice.

It is well established that the collection of age and sex data is epidemiologically significant for identifying disease distribution patterns. In this context, these variables are also essential for capturing the breadth of patient profiles managed in OMS. Another relevant factor to consider is the identification of the patient's systemic profile, which highlights the close relationship between OM and complex patients (Whitney et al. 2015), as well as the need to explore systemic diseases that can impact oral health (Porter et al. 2017; Bhalla et al. 2020). The identification of patients' harmful habits is also highly relevant, as it provides insight into primary and secondary prevention efforts, areas in which OM professionals are actively involved. An example of this is the role of tobacco and alcohol use as etiological factors for oral cancer (Bouvard et al. 2022).

The description of diagnoses in OM studies is crucial for understanding and exemplifying the extensive range of conditions that OM professionals are trained to recognise and manage. Some studies have demonstrated that most referrals do not include a provisional diagnosis (Navarro et al. 2001; Sardella et al. 2007; Villa et al. 2015), and when a diagnosis is provided, it is often incorrect (Sardella et al. 2007; Coppola et al. 2021). This reinforces the need for OM professionals and services. In this regard, it is important that studies describe diagnoses quantitatively and qualitatively in detail, rather than merely presenting them in aggregated form or indicating only the most prevalent diagnoses.

This also applies to procedures, including both diagnostic and treatment protocols. However, most studies in this review did not present this aspect of the scope of practice, even in an aggregated manner. Another fundamental aspect regarding patients treated in OMS is the need for follow‐up, which was not clearly demonstrated in any of the included articles. To recognise and establish the significance of these professionals and OMS, a clear demonstration of their scope of practice is essential. The heterogeneous availability of variables regarding the scope of OMS reflects the specific objectives proposed in each article at the time of its publication.

A solid characterisation of the clinical scope of OM practice within the body of evidence in the literature has the potential to enhance global understanding and recognition of the field, potentially reducing referral pathways. Furthermore, the global mapping of scopes may contribute to establishing training strategies for the education of specialists in the field. Lastly, through the demonstration of practices from various services, it will be possible to identify opportunities for international collaboration. This scoping review has limitations that should be considered. First, the lack of measurement of other ways OMS demonstrate their scope of practice, beyond the publication of scientific articles. Second, it is possible that other data not included in this review are relevant to demonstrating OMS practice. Finally, while demonstrating how OMS present their practices does not directly lead to increasing the recognition of the field, it highlights how future studies can address gaps in the existing literature.

Some studies specifically aimed to characterise the practice in OMS, while others addressed specific aspects of these practices. However, there are gaps in the demonstration of OMS practices, particularly regarding information on patient follow‐up, characterisation of the range of procedures (diagnostic and therapeutic), and documentation of patients' medical profiles. It is recommended that future studies aiming to characterise the clinical scope of OM include, when possible, the set of variables assessed in this review (File S5). For investigations focusing on specific aspects of clinical practice, efforts should be directed towards addressing the persistent gaps identified. This will contribute to emphasising the breadth and multidisciplinary nature of OM, ultimately leading OM to a wider global recognition.

## Author Contributions

**Camila Barcellos Calderipe:** conceptualization, methodology, data curation, writing – original draft, writing – review and editing, investigation, visualization, formal analysis. **Laura Borges Kirschnick:** conceptualization, methodology, data curation, investigation, writing – review and editing, visualization, formal analysis. **Alini Cardoso Soares:** conceptualization, writing – review and editing, visualization, formal analysis. **Ana Carolina Uchoa Vasconcelos:** conceptualization, visualization, writing – review and editing, formal analysis. **Marcio Ajudarte Lopes:** conceptualization, writing – review and editing, visualization, formal analysis. **Nathaniel Simon Treister:** conceptualization, writing – review and editing, formal analysis, visualization. **Alan Roger Santos‐Silva:** conceptualization, writing – review and editing, formal analysis, validation, writing – original draft, supervision.

## Conflicts of Interest

The authors declare no conflicts of interest.

## Supporting information


**File S1.** Personalised search strategies.

**File S2.** General features of the included studies.

**File S3.** Assessment of the availability of variables from oral medicine services.

**File S4.** Overview of the investigated variables according to the highest prevalences.

**File S5.** Minimum standard checklist for reporting the scope of clinical practice in oral medicine services.

## Data Availability

The data that support the findings of this study are available in the Supporting Information of this article.
