# Supplementary material for: How Oral Medicine Practice Is Reported: A Scoping Review of 114,971 Patients
Source: Oral Dis. 2025 Jul 2;31(12):3253–9. doi: 10.1111/odi.70017 (PMC12989044; doi:10.1111/odi.70017)
Supplement: Supplementary file 1 — File S1. Personalised search strategies. [file ODI-31-3253-s001.docx]

**Supplementary file 1**- Personalized search strategies.

| **Database** | **Search strategy**  (Search date: August 3^rd^, 2024) | **Results** |
| --- | --- | --- |
| PubMed | (“oral medicine”[Title/Abstract] OR stomatology[Title/Abstract]) **AND** (scope[Title/Abstract] OR “clinical practice”[Title/Abstract]) | 172 |
| Scopus | TITLE-ABS-KEY (“oral medicine” OR stomatology ) **AND** TITLE-ABS-KEY ( scope OR “clinical practice”) | 335 |
| Embase | (‘oral medicine’/de OR ‘stomatology’/de) **AND** (‘scope’/de OR ‘clinical practice’/de) | 69 |
| Web of Science | “oral medicine” OR stomatology (Topic) **AND** scope OR “clinical practice” (Topic) | 171 |
| LILACS | (estomatologia OR “medicina oral” OR “oral medicine” OR stomatology) **AND** (escopo OR “prática clínica” OR alcance OR “práctica clínica” OR scope OR “clinical practice”) | 78 |
| ProQuest | TI,AB(“oral medicine” OR stomatology) **AND** TI,AB (scope OR “clinical practice”) | 142 |
| Google Scholar | First 100 more relevant hits. No patents and no citations (“oral medicine” OR stomatology) **AND** (scope OR “clinical practice”) | 100 |
