# Supplementary material for: How Oral Medicine Practice Is Reported: A Scoping Review of 114,971 Patients
Source: Oral Dis. 2025 Jul 2;31(12):3253–9. doi: 10.1111/odi.70017 (PMC12989044; doi:10.1111/odi.70017)
Supplement: Supplementary file 2 — File S2. General features of the included studies. [file ODI-31-3253-s002.docx]

**Supplementary file 2-** General features of the included studies.

| **Author** (Year) | **Country** | **Service** | **Setting** | **Sample size** | **Period collected**  (years) | **Objective** | **Conclusions** |
| --- | --- | --- | --- | --- | --- | --- | --- |
|  |  |  |  |  |  |  |  |
| Farah et al.  (2007) | Australia | Hospital-based oral medicine and oral pathology clinic - School of Dentistry - University of Queensland | Hospital | 500 | 5 | To examine the **scope of practice**, **lesion** occurrence and utilization of referral-based hospital and private practice OMP services in Australia. | There is a relatively high occurrence of oral conditions, for which healthcare practitioners deem the diagnosis and management to be outside their scope of practice. This indicates that OMP services in Australia are crucial, and that the demand for these services is strong. |
|  | Australia | Private clinic oral medicine - Central Business District of Brisbane -  Queensland | Private  clinic | 1104 | 5 |  |  |
| Suarez & Clark (2007) | USA | Orofacial pain and oral medicine - School of dentistry - University of Southern California | Dental School | 1049 | 2 | To provide information on the **scope of OM practice**, to help practitioners create age-appropriate differential diagnoses, and to help dental school curriculum committees and graduate program directors assess their curricula to ensure they are including the full range of oral conditions in their programs. | This paper did report on what type of patient was seeking care and the data presented captured information about the need and demand for treatment. With this information, educators and clinicians can better understand what would constitute a minimum set of skills and basic knowledge needed to service patients in the private patient care sector. |
| Villa et al.  (2015) | USA | Division of oral medicine and dentistry - Brigham and Women’s Hospital - Harvard School of Dental Medicine - Harvard University | Hospital | 1043 | 3 | To characterize comprehensively the outpatient **OM practice** in a hospital-based setting within a tertiary care academic medical center, with particular emphasis on patient **demographic** characteristics, clinical **diagnoses**, and **referral** patterns. | Patients with oral conditions often see more than one doctor, before being referred to an OM expert and typically travel twice the distance to the expert compared with that between their home and the referring doctor. Equal efforts should be made to increase awareness of the importance of the specialty of OM among dentists, physicians, and the public. |
| Rodphon et al. (2020) | Thailand | Oral medicine - Faculty of dentistry- Mahidol University | Dental School | 540 | NR | To evaluate the **scope of practice**, **lesion** and **condition** occurrence, and **referral** patterns in patients who were referred for OM service at the Special Clinic, Faculty of Dentistry, MU, Thailand. | The results of this study will be beneficial for the education of dental practitioners both in the dental schools and for continuing education in the future. |
| Sun et al. (2020) | USA | Oral medicine - Penn Dental Medicine - University of Pennsylvania | Dental School | 1648 | 5 | To characterize OM **clinical practices** at the University of Pennsylvania, determine the importance of OM clinical services, and emphasize aspects of training for OM specialists. | Analysis of Penn OM clinical practices emphasize the breadth and multidisciplinary nature of OM services and importance of comprehensive postdoctoral training in all domains of OM to ensure appropriate provision of clinical services within the specialty. |
|  | USA | Oral medicine - University of Pennsylvania Health System - University of Pennsylvania | Hospital | 6337 | 5 |  |  |
| Bottomley et al. (1990) | USA | Oral medicine - School of  dentistry - Georgetown University | Dental School | 981 | 8 | To characterize the **distribution of patients referred** to a particular OM practice. To identify orofacial conditions that are apparent diagnostic and management dilemmas. Other goals include influencing perspective practice attitudes, implying a need for greater emphasis of these topics on educational curricula, suggesting areas for research concentrations, and making healthcare professions aware of the incidence of these conditions. | This study can be helpful in addressing educational and research needs of OM private practice in the future. Our data indicate that clinicians need improved expertise in the diagnosis and treatment of many oral conditions such as lichen planus, recurrent aphthous stomatitis, and fibromyalgia. |
| Ni Riordain et al. (2011) | Ireland | Oral medicine - Cork University Dental School and Hospital - University College Cork | Hospital | 378 | 1 | To examine patient **demographics**, **lesion** types and **referral** sources to CUDSH for OM services and secondly, to indicate factors that could improve the efficiency of the service provided. | Based on the results presented there appears to be a considerable demand for a service dealing with oral lesions and conditions, falling under the remit of OM, which other practitioners consider to be outside the scope of their practice. Studies of this nature should prove to be valuable in maximizing hospital resources at a time when economic factors are leading to increased financial pressure in health care funding. |
| Calcia et al. (2019) | Brazil | Oral medicine - Dental school - São Leopoldo Mandic School | Dental School | 175 | 2.25 | To evaluate the most diagnosed **lesions**, as well the main **epidemiological** characteristics of the patients attended at the oral medicine clinic of SL Mandic School - Rio de Janeiro in the period between 2014 and 2016. | Predilection for female patients with ages between 40 and 69 years was observed. Also, white-skinned declared patients in both genders represented majority of our sample. Epithelial pathology and soft tissue tumors were the most frequently group of lesions diagnosed on our clinic, with an especially increased incidence of fibrous hyperplasia. Finally, we found a high agreement between the initial and final diagnosis, highlighting the excellence of this service. |
| Friesen et al. (2019) | Canada | Oral medicine - Faculty of medicine and dentistry - University of Alberta | School of medicine and dentistry | 924 | 1 | To characterize and analyze **referral** patterns to a university-based outpatient OM clinic and to identify access-to-care issues for this referral patient population. | The referral patterns of dental and medical practitioners are similar, with mucosal lesions being the most common referral reason. In our study population, access to care was compromised by wait times and travel distances. These data may help support the future development of curricula for dental students and OM residents and inform the designing of continuing education courses of dentists and physicians. |
| Balkaran et al. (2021) | Trinidad  and Tobago | Oral medicine - Dental school - University of the West Indies | Dental School | 106 | 1 | To determine the **lesion** types and **referral** sources to the UWI OM Department and secondly, to examine the patient **demographics** and determine the meantime taken from patient referral to consultation. | Most referrals were from general dentists. The common conditions that were referred were oral white lesions and raised soft tissue lesions. Also, there is a need to reduce the wait time from the referral to the point that they are initially seen/examined at UWI. |
| Coppola et al. (2021) | Italy | Oral medicine - School of medicine - University of Naples Federico II | Medicine School | 583 | 1.83 | To analyze the **referral** process to the OM Unit in a university-based tertiary center in Southern Italy. | There is a growing demand for assistance in this field related to the high incidence of oral diseases and the crucial role that OM services play. There is a low grade of correct diagnosis by referral provider with the consequent need to implement curricula among dentistry and medical students. Finally, it is imperative that OM becomes even more of a reference for the medical community as well, to provide complete medical assistance to patients. |
| Han et al. (2022) | New  Zealand | Oral medicine- Faculty of dentistry - University of Otago | Dental School | 2533 | 6 | To investigate the **patterns of patients** managed at two OM service centers, with a focus on patient **demographics**, **diagnostic services used to make a diagnosis**, the spectrum of **orofacial conditions** encountered, and the **treatments** provided by OM services. | Most patients in OM clinics were 50 to 70-year-old women. Blood tests, imaging, and biopsy were the main clinical investigations. Most of the diagnoses were oromucosal diseases. The main treatments were self-care and corticosteroid prescriptions. These results provided insight into the range of orofacial conditions that were diagnosed and oral medicine services that were used. |
|  | China | Oral medicine - Weifang People’s Hospital | Hospital | 97070 | 6 |  |  |
| OMP: oral medicine and oral pathology; NR: not reported; OM: oral medicine; MU: Mahidol University Penn: University of Pennsylvania; USA: United States of America; CUDSH: Cork University Dental School and Hospital; UWI: University of the West Indies; SL: São Leopoldo; **Highlight: explicit proposal to evaluate the practice in OM services**; **Highlight**: **emphases within the scope of practice in OM**; Highlight: major conclusions. | | | | | | | |
