# Supplementary material for: How Oral Medicine Practice Is Reported: A Scoping Review of 114,971 Patients
Source: Oral Dis. 2025 Jul 2;31(12):3253–9. doi: 10.1111/odi.70017 (PMC12989044; doi:10.1111/odi.70017)
Supplement: Supplementary file 3 — File S3. Assessment of the availability of variables from oral medicine services. [file ODI-31-3253-s005.docx]

|  | **Referral source n** (%) | **Age n** (%) | | **Sex n** (%) | **Comorbidities n** (%) | **Harmful habits n** (%) | **Diagnosis n** (%) | **Procedures n** (%) | | **Follow-up n** (%) |
| --- | --- | --- | --- | --- | --- | --- | --- | --- | --- | --- |
|  |  | Mean | Range |  |  |  |  | Diagnosis | Therapeutics |  |
| **Presence** | **10**  (66.7) | **11**  (73.3) | **8**  (53.3) | **13**  (86.7) | **4** (26.7) | **3** (20.0) | **8**  (53.3) | **4**  (26.7) | **2**  (13.3) | **0**  (0.0) |
| **Unclear** | **0** (0.0) | **2** (13.3) | **0**  (0.0) | **0** (0.0) | **0**  (0.0) | **0** (0.0) | **7**  (46.7) | **5**  (33.3) | **3**  (20.0) | **2**  (13.3) |
| **Absence** | **5** (33.3) | **2**  (13.3) | **7**  (46.7) | **2**  (13.3) | **11** (73.3) | **12** (80.0) | **0** (0.0) | **6**  (40.0) | **10**  (66.7) | **13**  (86.7) |

**Supplementary file 3-** Assessment of the availability of variables from Oral Medicine Services.
