# Supplementary material for: How Oral Medicine Practice Is Reported: A Scoping Review of 114,971 Patients
Source: Oral Dis. 2025 Jul 2;31(12):3253–9. doi: 10.1111/odi.70017 (PMC12989044; doi:10.1111/odi.70017)
Supplement: Supplementary file 4 — File S4. Overview of the investigated variables according to the highest prevalences. [file ODI-31-3253-s003.docx]

**Supplementary file 4**- Overview of the investigated variables according to the highest prevalences.

| **Author (Year)** | **Sample size** | **Referral source** (n/%) | **Age (years)** | | **Sex** | | **Comorbidities** (n/%) | **Harmful habits** (n/%) | **Diagnosis** (n/%) | **Procedures** | | **Follow-up** |
| --- | --- | --- | --- | --- | --- | --- | --- | --- | --- | --- | --- | --- |
|  |  |  | **Mean** | **Range** | **Female** (n/%) | **Male** (n/%) |  |  |  | **Diagnosis** (n/%) | **Therapeutics** (n/%) |  |
| Bottomley et al. (1990) | 981 | NI | 47.7 | 3-90 | 673/68.6 | 308/31.4 | NI | NI | Oral lichen planus 193/19.7 | NI | NI | NI |
| Farah et al.  (2007) | 500 | Internal* 242/48.4 | 51.1 | 1-90 | 279/55.8 | 221/44.2 | Cardiovascular 188/18.2 | Smokers 124/24.8 | Epithelial hyperplasia/keratosis 96/11.5 | Biopsy** 92/18.4 | Pharmacology  180/36.6 | NI |
|  | 1.104 | Dentist 905/82.0 | 50.2 | 0,3-94 | 666/60.3 | 438/39.7 | Allergy  397/29.2 | Smokers 254/23.0 | Epithelial hyperplasia/keratosis 120/10.2 | Biopsy** 213/19.3 | Pharmacology  561/50.8 | NI |
| Suarez & Clark (2007) | 1.049 | NI | +64^a^ | NI | 710/67.7 | 339/32.3 | NI | NI | Temporomandibular disorder capsulitis 163/15.5 | NI | NI | NI |
| Ni Riordain et al.  (2011) | 378 | Dentist 279/73.8 | 46.6 | NI | 248/65.6 | 130/34.4 | NI | NI | White lesion^b^ 65/17.2 | NI | NI | NI |
| Villa et al.  (2015) | 1.043 | Medical 695/66.0 | 56 | 15-96 | 666/63.8 | 377/36.2 | NI | NI | Oral lichen planus 124/11.9 | Biopsy** 188/18.0 | Pharmacology^c^ 375/35.9 | "Within 6 months of the initial visit, approximately half the patients  (444 of 1043; 43.6%) returned for follow-up."^d^ |
| Calcia et al. (2019) | 175 | NI | Female 60 to 69^e^ 25/14.3  Male 60 to 69^e^ 14/8.0 | NI | 110/63.0 | 65/37.0 | NI | NI | Fibrous hyperplasia 11/8.0 | NI | NI | NI |
| Friesen et al. (2019) | 924 | Dentist  752/81.4 | 56.6 | 4-101 | 564/61.0 | 360/39.0 | NI | NI | Immune-mediated^f^ 265/28.7 | NI | NI | NI |
| Rodphon et al. (2020) | 540 | Dentist 413/76.4 | 54 | 20-90 | 410/76.0 | 130/24.0 | NI | NI | Oral lichen planus/oral lichenoid lesion^f^ 196/37.7 | Biopsy** 143/26.5 | NI | NI |
| Sun et al. (2020) | 1.648 | NI | NI | NI | NI | NI | Human Immunodeficiency Virus 252/15.3 | NI | Orofacial pain^g^ 4373/45.0 | Biopsy**^g^ 375/59.3 | NI | NI |
|  | 6.337 | NI | NI | NI | NI | NI | Cardiovascular 2465/38.9 | NI |  |  | NI | NI |
| Balkaran et al. (2021) | 106 | Dentist 48/45.3 | 47.1 | NI | 64/60.4 | 42/39.6 | NI | NI | Oral lichen planus 17/16.0 | NI | NI | "Nearly 22 % of patients had a second follow-up visit documented in their respective files, with a mean time of 42.9 network days."^d^ |
| Coppola et al. (2021) | 583 | Dentist 366/62.9 | 56.6 | NI | 365/62.6 | 218/37.4 | NI | Alcohol consumption 368/63.1 | Oral lichen planus 164/28.1 | Blood test 31/13,7^h^ | NI | NI |
| Han et al. (2022) | 2.533 | Medical 696/27.5 | 53.6 | 2-92 | 1799/71.0 | 734/29.0 | NI | NI | Temporomandibular disorder  ^i^/21.1 | Blood test ^i^/22.1 | Self-care instructions and education ^i^/36.6% | NI |
|  | 97.070 | Medical (29.6%) | 51.2 | 0.08-96 | 54845/56.5 | 42225/43.5 | NI | NI | Temporomandibular disorder  ^i^/9.3 | Blood test ^i^/25.4 | Pharmacology ^i^/42.3% | NI |
| *:from various clinics from within the hospital itself (in which 53.07% undergraduate); **:in some cases it may have been treatment; NI: not informed; Unclear information: ^a^:19.6% were over 64 years of age; ^b^: the diagnosis was not reported.^c^: only one medication was mentioned; ^d^: limited information on patient return visits records. ^e^:most patients were between these ages; ^f^: grouped diagnoses; ^g^:no clear attribution for each of the separate OMS; ^h^: examination request was made outside of the OMS; ^i^: without the indication of the absolute number.   Some variables do not match the number of participants; for more accurate information, it is necessary to access the original article. | | | | | | | | | | | | |
