# Supplementary material for: How Oral Medicine Practice Is Reported: A Scoping Review of 114,971 Patients
Source: Oral Dis. 2025 Jul 2;31(12):3253–9. doi: 10.1111/odi.70017 (PMC12989044; doi:10.1111/odi.70017)
Supplement: Supplementary file 5 — File S5. Minimum standard checklist for reporting the scope of clinical practice in oral medicine services. [file ODI-31-3253-s004.docx]

| **Supplementary file 5**- Minimum standard checklist for reporting the scope of clinical practice in Oral Medicine services. | |  |
| --- | --- | --- |
| **Variables** | **Categories** | |
| Referral source | Example: dentist/physician/self-referral...; | |
| Mean age | In years; | |
| Age range |  |  |
| Sex | Female/male; | |
| Comorbities | Example: no/yes - quantity - type (s)*; | |
| Harmful habits | Example: no/yes (current use/former use) - quantity - type (s)*; | |
| Diagnosis | Example: quantity - type (s)*; | |
| Diagnostic procedures | Example: quantity - type (s)*; | |
| Therapeutic procedures | Example: quantity - type (s)*; | |
| Follow-up | Example: number of consultations - follow-up (in months) - progress of the consultations. | |
| * provide the individual naming/counting, even if it is necessary to group them in order to present the results. | | |
